# Inflammation-Associated Long Non-Coding RNAs (lncRNAs) in Chronic Viral Hepatitis-Associated Hepatocellular Carcinoma

**DOI:** 10.5146/tjpath.2024.13593

**Published:** 2025-01-31

**Authors:** Burcin Pehlivanoglu, Anil Aysal, Cihan Agalar, Tufan Egeli, Mucahit Ozbilgin, Tarkan Unek, Ilkay Tugba Unek, Ilhan Oztop, Safiye Aktas, Ozgul Sagol

**Affiliations:** Department of Molecular Pathology, Dokuz Eylul University, Graduate School of Health Sciences, İzmir, Türkiye; Department of Pathology, Dokuz Eylul University, Faculty of Medicine, İzmir, Türkiye; Department of General Surgery, Dokuz Eylul University, Faculty of Medicine, İzmir, Türkiye; Department of Medical Oncology, Dokuz Eylul University, Faculty of Medicine, İzmir, Türkiye; Department of Basic Oncology, Dokuz Eylul University, Institute of Oncology, İzmir, Türkiye

**Keywords:** Prognosis, Hepatocellular carcinoma, lncRNA, Inflammation, Chronic viral hepatitis, Cirrhosis

## Abstract

***Objective: *** This study aimed to identify the expression profile and prognostic significance of inflammation-associated lncRNAs in chronic viral hepatitis (CVH) and CVH-associated hepatocellular carcinoma (CVH-HCC).

***Material and Methods:*** In the first step, lncRNA expression analysis was performed by real-time polymerase chain reaction (RT-PCR) using an array panel of 84 inflammation-associated lncRNAs in 48 formalin-fixed paraffin-embedded (FFPE) tissue samples (12 CVH-HCC, 12 peritumoral cirrhotic parenchyma, 12 nontumoral cirrhotic CVH parenchyma, 12 normal liver samples). In the second step, 7 lncRNAs (*DLEU2, HOTAIR, LINC00635, LINC00662, RP11-549J18.1, SNHG16 *and* XIST*) were chosen for RT-PCR assay testing in 72 samples (24 CVH-HCC, 24 peritumoral cirrhotic parenchyma, 24 nontumoral cirrhotic CVH parenchyma samples).

***Results: *** Fifty-six inflammation-associated lncRNAs were significantly up-regulated in the peritumoral cirrhotic parenchyma compared to the normal liver. Expression of 71 lncRNAs was significantly higher in peritumoral cirrhotic parenchyma compared to cirrhotic CVH parenchyma. *DLEU2* and *SNHG16* were up-regulated both in the tumor and peritumoral cirrhotic parenchyma compared to cirrhotic CVH parenchyma. Expression of *LINC00662* was significantly higher in CVH-HCC than in cirrhotic CVH parenchyma. Expression of *XIST* was also increased in both tumor and peritumoral parenchyma samples, albeit without statistical significance. No significant association was found between lncRNA expressions and survival.

***Conclusion:*** Inflammation-associated lncRNAs *DLEU2, SNHG16, LINC00662,* and *XIST* are candidate diagnostic biomarkers in CVH-HCC. More evidence is needed to prove their utility as prognostic markers.

## INTRODUCTION

In chronic liver inflammation, such as in chronic viral hepatitis (CVH), recurrent cellular injury and regeneration cycles cause accumulation of mutations in hepatocytes and activation of inflammation-associated cellular pathways, eventually resulting in the formation of hepatocellular carcinoma (HCC). Viral infections may cause changes in both coding and noncoding regions of the host genome. Long non-coding RNAs (lncRNAs) are non-coding transcripts composed of >200 nucleotides. Recently, lncRNAs *NEAT1*, *lnc-DILC, lnc-PANDA*, *lnc-TCF7, MALAT-1, lncRNA 00607* and *lncRNA AX800134* have been found to contribute to HCC development through inflammatory pathways (1–7). However, limited data is available on the possible pathogenetic role of lncRNAs in CVH-associated HCC (CVH-HCC). To the best of our knowledge, inflammation-associated lncRNA expression profile has not been investigated in detail in patients with CVH-HCC. Therefore, in this study, we aimed to investigate the pathogenetic and prognostic effects of inflammation-associated lncRNAs in CVH-HCC, and to evaluate the association between expression of inflammation-associated lncRNAs and histopathological features.

## MATERIAL and METHODS

The study protocol was approved by the institutional ethics committee (2019/05-70).

### Case Selection

The study group was composed of 48 consecutive cirrhotic patients with CVH (n=24) and/or CVH-HCC (n=24) who had undergone liver transplantation without prior interventional treatment. In addition to the resection specimens of these 48 patients, for the first step, 12 normal liver tissue biopsy samples were also determined to serve as a control group. Only CVH or CVH-HCC cases were included in the second step.

### Collection of Clinicopathological Data

Clinicopathological data were retrieved from hospital records. Hematoxylin-eosin stained slides of the cases were re-evaluated for the selection of appropriate tissue blocks for molecular analysis, and to assess the severity and type of inflammation.

### Real-time Polymerase Chain Reaction (RT-PCR) on Formalin-Fixed Paraffin-Embedded (FFPE) Tissue Samples

Supplementary Table I PDF file supplied by authors.

Five sections of 7 microns were taken from the selected blocks for RT-PCR. First, inflammation-associated lncRNA expression levels were measured in 48 samples using a commercially available panel that includes 84 inflammation-associated lncRNAs (*Human Inflammatory Response and Autoimmunity RT2 lncRNA PCR Array, QIAGEN, Germany*) (Supplement Table I). Samples were grouped as follows: group 1: 12 CVH-HCC samples, group 2: 12 peritumoral cirrhotic parenchyma samples, group 3: 12 cirrhotic CVH samples, and group 4: 12 normal liver tissue samples.

Subsequently, 7 lncRNAs (*DLEU2, HOTAIR, LINC00635, LINC00662, RP11-549J18. 1, SNHG16, XIST*) (*RT² QPCR Primer Assay, Qiagen, Germany*) showing different expression levels with >2 or <-2-fold change in at least 3 group comparisons were further analyzed by assay testing. An inclusive approach was adopted in this random selection, i.e., statistical significance was not sought, considering the limited number of cases in each group. In the assay step, expression levels of these lncRNAs were assessed in 72 samples (24 CVH-HCCs, 24 peritumoral cirrhotic parenchyma, and 24 cirrhotic CVH parenchyma). Glyceraldehyde 3-phosphate dehydrogenase (*GAPDH*) was used as the reference gene in assay testing.

RNA extraction was done according to the manufacturer’s recommendations using a kit for FFPE tissues (*RNEASY FFPE Kit, Qiagen, Germany*). Then, cDNA synthesis and preamplification were performed using the *RT² PREAMP cDNA Synthesıs Kit (Qiagen, Germany*) prior to preparation for PCR. *RT2 SYBR Green Mastermix* (Qiagen, Germany) was used for RT-PCR sample preparation.

### Assessment of the Findings and Statistical Analysis

Data obtained via RT-PCR analysis (i.e., inflammation-associated lncRNA expression levels) were analyzed online using the ***ΔΔ*** Ct method at https://geneglobe.qiagen.com/tr/analyze. Three tumor samples and three normal liver samples in the RT-array step were excluded from analysis due to poor RNA quality. When comparing lncRNA expression levels, >2 and <-2 fold change was considered as up-regulation and down-regulation, respectively. The association between the expressions of *DLEU2, HOTAIR, LINC00635, LINC00662, RP11-549J18. 1, SNHG16, XIST* and clinicopathological parameters were statistically analyzed using SPSS ver. 24 (*IBM, USA*). The chi-square test was used to compare categorical variables and the Kruskal-Wallis test was used for comparisons between >2 groups. Dunn’s test was used to further demonstrate the significant differences between the subgroups. Overall survival (OS) was defined as the time period between the date of the operation and death for any reason or last follow-up date. The time from transplantation to the death of the patient because of the disease or to the last follow-up date represented disease-specific survival (DSS). In the CVH-HCC group, survival analysis was performed using the Kaplan-Meier method, and the Log-rank test was used to compare survival between ≥2 groups. The Holm adjustment was used for pairwise comparisons in survival analysis. Four patients who died within the post-operative 30 days were excluded from the survival analysis. p < 0.05 was considered statistically significant for all analyses.

## RESULTS

### Clinicopathological Characteristics

All cases had histopathologically confirmed cirrhosis.

Only 2 cases with CVH-HCC were female (M:F = 11). The median age was 58 ± 4.85 years (range 48-67 years). The etiologic agent was hepatitis B virus (HBV) in the majority (n=21, 87.5%), while the remaining 3 patients (12.5%) had chronic hepatitis C. Six of the HBV positive cases had hepatitis D virus (HDV) co-infection. Mean tumor diameter was 3.4 ± 1.56 cm, and 11 patients (45.8%) had multifocal HCC. About two thirds (n=16, 66.7%) had grade 2 tumors, and 4, 1, and 3 patients had grade 1, 3, and 4 tumors, respectively. pT stage was determined as pT1 in 13, pT2 in 7, pT3 in 1, and pT4 in 3 patients. Only 1 patient with CVH-HCC had lymph node metastasis; however, venous invasion was observed in 6 cases (25%). Portal vein thrombosis was noted in one patient.

Inflammatory cells were often scarce in HCC foci: only 4 had substantial and 3 had moderate intratumoral inflammation while 12 (50%) had mild intratumoral inflammation and the remaining 5 had only scattered few inflammatory cells within the tumor. The majority had neutrophil predominant mixed inflammation (n=15, 62.5%). Inflammatory activity was more prominent in peritumoral cirrhotic parenchyma and all had mixed peritumoral inflammatory infiltration (Table 1).

**Table 1:** Comparison of the clinicopathological features in chronic viral hepatitis associated hepatocellular carcinoma (CVH-HCC) vs. chronic viral hepatitis (CVH) groups

	**CVH-HCC (n=24)**	**CVH (n=24)**	**p value**
Gender Female/Male	2/22	17/7	0.064
Median age	58 ± 4.85 years (range 48-67)	50 ± 12.25 years (range 31-70)	**0.011**
Etiology HBV HCV HDV co-infection	21 (87.5%) 3 (12.5%) 6/21 cases with HBV	22 (91.7%) 2 (8.3%) 10/22 cases with HBV	>0.05
Degree of parenchymal/portal inflammation Mild Moderate Severe	4 (16.6%) 12 (50%) 8 (33.3%)	1 (4.1%) 10 (41.7%) 13 (54.2%)	0.2
Type of parenchymal/portal inflammation Mixed with abundant lymphocytes, plasmacytes and neutrophils Neutrophil predominant mixed Lymphoplasmacytic predominant with occasional neutrophils Lymphoplasmacytic predominant with rare neutrophils	8 (33.3%) 6 (25%) 5 (20.8%) 5 (20.8%)	6 (25%) 6 (25%) 8 (33.3%) 4 16.6%)	0.78

The CVH group consisted of 17 men and 7 women (M: F = 2.42). The median age was 50 ± 12.25 years (range 31-70 years). Only 2 patients had chronic hepatitis C while the majority (n=22, 91.7%) had chronic hepatitis B. Ten patients (41.7%) had HDV co-infection. More than half (n=13, 54.2%) had severe parenchymal/portal inflammation, 10 (41.7%) had moderate inflammation, and the remaining 1 patient had mild inflammation. In 12 patients, the predominant inflammatory cell type was lymphoplasmacytic but neutrophils were notable or predominant in 12 patients (Table 1).

Patients with HCC were significantly older than CVH cases (median 58 ± 4.85 vs. 50 ± 12.25, p=0.011). No other significant difference was found between the two groups.

## RT-Array Panel Results

Supplementary Table II PDF file supplied by authors.

Numerous lncRNAs were differentially expressed in the four groups, albeit some without statistical significance. Fifty-six inflammation-associated lncRNAs were significantly up-regulated in the peritumoral cirrhotic parenchyma compared to normal liver, and while 31 out of the 56 were expressed significantly lower in the tumor, none were significantly differentially expressed between the tumor and the normal liver. In contrast, only *SNHG11* and *XIST *were significantly differentially expressed in cirrhotic CVH than in the normal liver and their expressions were decreased. Moreover, expression of 71 lncRNAs was significantly higher in peritumoral cirrhotic parenchyma compared to cirrhotic CVH parenchyma (p<0.05). Among these, lncRNAs *RP11-473I1.10* and *RP11-819C21.1* were also significantly up-regulated in the tumor (compared to CVH) (fold change 2.45 and 3.47, p=0.03 and 0.04, respectively). There was fold change difference in the expression of several inflammation-associated lncRNAs when the tumor group and normal liver (control group) were compared, but none reached statistical significance (Supplement Table II).

### Assay Results

**Figure 1:**
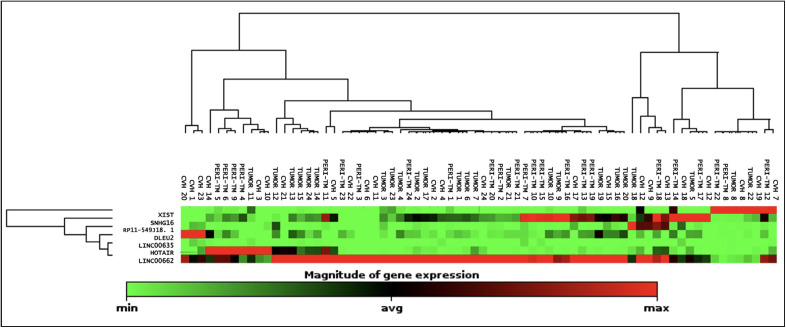
Clustergram showing the assay results

Assay testing revealed that *DLEU2* and *SNHG16* were up-regulated both in the tumor and peritumoral cirrhotic parenchyma compared to cirrhotic CVH parenchyma samples (15.98 and 10.34 fold, p=0.009 and 0.12 for *DLEU2*, and 15.7 and 19.4 fold, p= 0.017 and 0.012 for *SNHG16*, respectively). Although *LINC00662* was expressed in all tumoral and nontumoral tissues, its expression was higher in the tumor and peritumoral parenchyma than in cirrhotic CVH parenchyma, but the difference between peritumoral and CVH parenchyma did not reach statistical significance (11.87 and 11.05 fold, p=0.012 and 0.16, respectively). Expression of *XIST* was also increased in both tumor and peritumoral parenchyma samples, albeit without statistical significance (2.10 and 3.60 fold, p=0.41 and 0.36, respectively) (Figure 1, Table 2).

**Table 2:** Results of the assay testing

**lncRNA**	**CVH-HCC vs. CVH**		**Peritumoral parenchyma vs. CVH**	
	**Fold Regulation**	**p value**	**Fold Regulation**	**p value**
*DLEU2*	15.98	**0.009**	10.34	0.12
*HOTAIR*	2.76	0.14	1.98	0.26
*LINC00635*	-1.07	0.15	-1.08	0.24
*LINC00662*	11.87	**0.012**	11.05	0.16
*RP11-549J18. 1*	1.33	0.48	2.03	0.19
*SNHG16*	15.70	**0.017**	19.40	**0.012**
*XIST*	2.10	0.41	3.60	0.368

**CVH-HCC: **Chronic viral hepatitis associated hepatocellular carcinoma, **CVH:** Chronic viral hepatitis.

### Association Between lncRNA Expression and Histopathological Features in CVH-HCC

*LINC00662 *and *SNHG16* were expressed in all tumor samples. None of the well-differentiated (grade 1) CVH-HCCs expressed *XIST* (0/4 vs. 13/20, p=0.02), and tumoral *XIST* expression was significantly associated with the presence of venous invasion (6/6 vs. 7/11; p=0.011).

Of the 9 cases with dysplastic nodules, 7 had peritumoral *SNHG16* expression, albeit without statistical significance (p=0.062). Peritumoral *XIST* expression was significantly associated with the presence of neutrophil predominant inflammation in peritumoral cirrhotic parenchyma, and it was significantly more common in peritumoral cirrhotic parenchyma with neutrophil predominant inflammation than in peritumoral cirrhotic parenchyma with lymphoplasmacytic predominant inflammation (6/6 vs. 2/10, p=0.007, Kruskal-Wallis and Dunn tests).

In addition, despite the limited number of patients with chronic hepatitis C infection, *XIST* expression was significantly associated with hepatitis C virus (HCV) positivity in the entire study group (4/5, p=0.029).

### Association Between Survival and lncRNA Expression in CVH-HCC

Median follow-up time was 91.28 ± 37.11 months (range 9.40-140.48 months). Eight patients died during follow-up; however, the cause of death was available to the authors in only 4: 1 patient died of pneumonia, 1 died of chronic liver rejection and late-onset sepsis, 1 died of metachronous lung cancer, and the remaining patient was the only one who died of disease (due to HCC metastasis). Mean OS was 105.13 ± 10.34 months (range 84.85-125.41 months). Five-year OS and DSS was 80% (95% CI 64.3%-99.6%) and 93.8% (95% CI 82.6%-100%), respectively.

There were some associations between the studied lncRNAs and survival that did not reach statistical significance. CVH-HCC patients with tumoral *DLEU2 *expression tended to have a better prognosis compared to the patient without tumoral *DLEU2* expression [106.46 ± 10.81 (95% CI 85.26-127.67) vs. 93.83 ± 0 months (one patient only), p=0.49]. CVH-HCC patients with peritumoral *LINC00635* expression had shorter mean OS [79.97 ± 11.98 (95% CI 56.49-103.45) vs. 112.73 ± 12.60 months (95% CI 88.03-137.44), p=0.11]. CVH-HCC cases with peritumoral *XIST* expression [113.8 ± 13.6 (95% CI 87.02-140.62) vs. 94.51 ± 14.91 months (95% CI 65.28-123.73), p=0.26] had longer mean OS. Cases with tumoral *HOTAIR* expression showed longer mean OS [108.90 ± 11.18 (95% CI 86.99-130.81) vs. 70.27 ± 13.54 months (95% CI 43.73-96.81), p=0.25]. Lastly, patients with tumoral *RP11-549J18.1* expression tended to have a better prognosis [106.75 ± 12.94 (95% CI 81.38-132.13) vs. 85.39 ± 10.77 months (95% CI 64.28-106.51), p=0.6].

Recurrence occurred in 3 patients (as metastatic bone disease in 2 and recurrent HCC with bone metastasis in the other), with a mean time to recurrence of 29.5 ± 10.92 months (median 29.16). Microvascular invasion was present in 2 of the 3 patients with recurrence. Three patients with recurrence expressed *LINC00635* in the peritumoral cirrhotic parenchyma. Moreover, of the 3 cases that did not express *HOTAIR* in the tumor tissue, 2 had recurrent disease.

## DISCUSSION

In this study, we investigated the expression of inflammation-associated lncRNAs in FFPE tissue samples of cirrhotic CVH-HCC and we observed several significant associations.

Using the inflammation-associated lncRNA RT-array panel, we found that there were many inflammation-associated lncRNAs that were differentially expressed in tumoral, peritumoral, and nontumoral cirrhotic liver parenchyma and normal liver samples. Among them, *SNHG11* and *XIST* were significantly down-regulated in cirrhotic CVH than in the normal liver. Down-regulation of *SNHG11* and *XIST* has previously been associated with ongoing inflammatory processes, supporting our findings (8–10). Curiously, these two lncRNAs have been shown to have tumor-promoting effects on hepatocellular carcinoma cells (11,12). The fact that *SNHG11* and *XIST* were significantly up-regulated (21.54 fold and 8.85 fold, respectively) in the peritumoral cirrhotic parenchyma compared to non-tumoral CVH parenchyma indicates an expression level dependent oncogenic (pro-proliferation) effect.

Assay testing has shown that *SNHG16*, which has been shown to interact with the NF‐κB pathway (13), was significantly up-regulated both in the tumor samples and peritumoral cirrhotic parenchyma compared to cirrhotic CVH parenchyma samples (15.7 and 19.4 fold). *SNHG16* was expressed in all tumor samples. Moreover, 7 of the 9 cases with dysplastic nodules showed peritumoral *SNHG16* expression. *SNHG16* was also significantly upregulated in the peritumoral parenchyma samples, compared to both CVH parenchyma and normal liver tissue in the RT-array test. These findings suggest that *SNHG16* up-regulation plays a major role in hepatocarcinogenesis, supporting the recent studies reporting that *SNHG16* interaction with microRNAs/cellular pathways contributes to development and proliferation of hepatocellular carcinoma cells (13–15).

*SNHG16* has also been implicated to be involved in resistance to sorafenib, a drug that is widely used for treating hepatocellular carcinoma (16–18), and has been described as a potential biomarker of unfavorable prognosis in a recent meta-analysis of 5 studies (19). However, we did not find any significant association between tumoral *SNHG16* expression and conventional negative prognostic parameters. While this may be partly due to the small number of the patients, it should also be kept in mind that the meta-analysis included only 5 studies. Therefore, the value of *SNHG16* as an independent prognostic factor in hepatocellular carcinoma should be further tested.

*DLEU2* was up-regulated in tumoral and peritumoral tissues compared to cirrhotic CVH parenchyma samples (15.98 and 10.34 fold). However, the difference in *DLEU2* expression between peritumoral and CVH parenchyma samples did not reach statistical significance despite 10.34 fold increase, in contrast to the array step, in which we observed a statistically significant up-regulation in the peritumoral parenchyma compared to CVH parenchyma and the normal liver. *DLEU2* has been shown to be up-regulated in HCC tissue and induce the proliferation in hepatocellular carcinoma cells in a recent study, supporting our findings (20). On the other hand, we did not observe any significant association between *DLEU2* expression and clinicopathological features including survival. The only patient who died of the disease had tumoral *DLEU2* expression. However, the patient also had vascular invasion and multiple tumor foci, indicating that *DLEU2* expression cannot be the sole culprit of death in this patient. In a recent study, Fu et. al (21) have claimed that *DLEU2* is upregulated in HCC tissue and its expression is associated with metastatic disease but the authors failed to demonstrate *DLEU2* as an independent prognostic factor in multivariate analyses. Of note, Salerno et al. (22) have demonstrated that HBx protein of HBV binds to *DLEU2* leading to cancer-related transcription in the host liver. However, we did not find a significant association between *DLEU2* expression and HBV positivity in our study group.

Expression of *XIST* was also increased in both tumor and peritumoral parenchyma samples, albeit without statistical significance (2.10 and 3.60 fold). None of the well-differentiated CVH-HCCs expressed *XIST,* and tumoral *XIST *expression was significantly associated with the presence of venous invasion. Although these findings indicate that tumoral *XIST* expression may negatively affect survival, we did not find any significant difference regarding survival in cases with and without tumoral *XIST* expression. This may be attributed to the small number of patients. On the other hand, while *XIST* expression has been reported to be associated with poor prognosis in several cancer types, it has also been claimed to have the potential to act as a tumor suppressor in certain scenarios (23,24), further questioning its prognostic value. Curiously, we found that *XIST* expression was significantly associated with HCV positivity in the entire study group, in consistence with the previous studies reporting that HCV core protein causes upregulation of miR-92b, which interacts with lncRNA *XIST* (25–27).

*HOTAIR* expression has recently been reported to be associated with advanced pT stage tumors, and high-expression level has been found to indicate poorer prognosis without any significant association with age, gender, or tumor size (28). However, our results challenge this meta-analysis as we observed that CVH-HCC patients with tumoral *HOTAIR* expression tended to have a longer mean survival and 2 of the 3 cases that did not show tumoral *HOTAIR* expression had recurrent disease.

Although *LINC00662* was expressed in all samples, its expression was higher in the tumor and peritumoral parenchyma than in cirrhotic CVH parenchyma. This finding challenges a previous study that claimed *LINC00662* upregulation to be a biomarker of poor prognosis (29). Naturally, there may be populational differences in expression of *LINC00662* in CVH-HCC. However, currently the significance of *LINC00662* expression seems to be more of pathogenetic, rather than being a prognostic factor.

There was peritumoral *LINC00635* expression in the 3 patients with recurrence. Moreover, although the difference was not statistically significant, CVH-HCC patients with peritumoral *LINC00635* expression had shorter mean overall survival. There is only one study on *LINC00635* expression in HCC, and in that study, serum *LINC00635* expression has been reported to be associated with lymph node metastasis, advanced stage, and worse OS (30). Naturally, serum levels cannot determine the exact source of *LINC00635*, i.e., tumor or peritumoral tissue.

The most challenging limitation of this study was RNA extraction from the FFPE tissues, as the destructive effect of formalin on nucleic acids is widely known. Although six samples had to be omitted from statistical analysis of the RT-array step due to poor RNA quality, all samples could successfully be analyzed in assay analysis. Also, using FFPE tissue samples provided the advantage of simultaneous histopathological evaluation.

In conclusion, chronic inflammation causes changes in expression levels of lncRNAs in cirrhotic liver parenchyma. The fact that some of these lncRNAs are also up- or down-regulated in CVH-HCC suggests that they may be involved in all stages of hepatocarcinogenesis. Inflammation associated lncRNAs are candidate diagnostic biomarkers in CVH-HCC. However, verification in larger patient groups are needed to evaluate whether they have true prognostic utility.

## Funding

This study was supported by the Research Fund of the Dokuz Eylul University. Project Number: 2021.KB.SAG.019.

## Conflict of Interest

The authors have no conflicts of interest to declare.

## References

[ref-1] Wang Shuai, Zhang Qian, Wang Qinlan, Shen Qicong, Chen Xiang, Li Zhenyang, Zhou Ye, Hou Jin, Xu Bowen, Li Nan, Cao Xuetao (2018). NEAT1 paraspeckle promotes human hepatocellular carcinoma progression by strengthening IL-6/STAT3 signaling. Oncoimmunology.

[ref-2] Wang Xue, Sun Wen, Shen Weifeng, Xia Mingyang, Chen Cheng, Xiang Daimin, Ning Beifang, Cui Xiuliang, Li Hengyu, Li Xiaofeng, Ding Jin, Wang Hongyang (2016). Long non-coding RNA DILC regulates liver cancer stem cells via IL-6/STAT3 axis. J Hepatol.

[ref-3] Peng Chuanhui, Hu Wendi, Weng Xiaoyu, Tong Rongliang, Cheng Shaobing, Ding Chaofeng, Xiao Heng, Lv Zhen, Xie Haiyang, Zhou Lin, Wu Jian, Zheng Shusen (2017). Over Expression of Long Non-Coding RNA PANDA Promotes Hepatocellular Carcinoma by Inhibiting Senescence Associated Inflammatory Factor IL8. Sci Rep.

[ref-4] Wu Jun, Zhang Jun, Shen Bin, Yin Kai, Xu Jianwei, Gao Wencan, Zhang Lihong (2015). Long noncoding RNA lncTCF7, induced by IL-6/STAT3 transactivation, promotes hepatocellular carcinoma aggressiveness through epithelial-mesenchymal transition. J Exp Clin Cancer Res.

[ref-5] Huang Mingyan, Wang Huamin, Hu Xiang, Cao Xuetao (2019). lncRNA MALAT1 binds chromatin remodeling subunit BRG1 to epigenetically promote inflammation-related hepatocellular carcinoma progression. Oncoimmunology.

[ref-6] Sun Qi-Man, Hu Bo, Fu Pei-Yao, Tang Wei-Guo, Zhang Xin, Zhan Hao, Sun Chao, He Yi-Feng, Song Kang, Xiao Yong-Sheng, Sun Jian, Xu Yang, Zhou Jian, Fan Jia (2018). Long non-coding RNA 00607 as a tumor suppressor by modulating NF-κB p65/p53 signaling axis in hepatocellular carcinoma. Carcinogenesis.

[ref-7] Zuo Kai, Kong Li, Xue Dong, Yang Yanyan, Xie Linlin (2018). The expression and role of lncRNA AX800134 in hepatitis B virus-related hepatocellular carcinoma. Virus Genes.

[ref-8] Song Tian-Jiao, Ke Jun, Chen Feng, Zhang Jiu-Yun, Zhang Chun, Chen Hong-Yi (2022). Effect of SNHG11/miR-7-5p/PLCB1 Axis on Acute Pancreatitis through Inhibiting p38MAPK Pathway. Cells.

[ref-9] Shen Conglin, Li Jialu (2021). LncRNA XIST silencing protects against sepsis-induced acute liver injury via inhibition of BRD4 expression. Inflammation.

[ref-10] Shenoda Botros B., Ramanathan Sujay, Gupta Richa, Tian Yuzhen, Jean-Toussaint Renee, Alexander Guillermo M., Addya Sankar, Somarowthu Srinivas, Sacan Ahmet, Ajit Seena K. (2021). Xist attenuates acute inflammatory response by female cells. Cell Mol Life Sci.

[ref-11] Dong Zhidan, Yang Juan, Zheng Fuchang, Zhang Yao (2020). The expression of lncRNA XIST in hepatocellular carcinoma cells and its effect on biological function. J BUON.

[ref-12] De Giorgi Valeria, Monaco Alessandro, Worchech Andrea, Tornesello Marialina, Izzo Francesco, Buonaguro Luigi, Marincola Francesco M., Wang Ena, Buonaguro Franco M. (2009). Gene profiling, biomarkers and pathways characterizing HCV-related hepatocellular carcinoma. J Transl Med.

[ref-13] Hu Yi-Lin, Feng Ying, Chen Yu-Yan, Liu Jia-Zhou, Su Yang, Li Peng, Huang Hua, Mao Qin-Sheng, Xue Wan-Jiang (2020). SNHG16/miR-605-3p/TRAF6/NF-κB feedback loop regulates hepatocellular carcinoma metastasis. J Cell Mol Med.

[ref-14] Xie Xuhua, Xu Xiaopei, Sun Changyu, Yu Zujiang (2019). Long intergenic noncoding RNA SNHG16 interacts with miR-195 to promote proliferation, invasion and tumorigenesis in hepatocellular carcinoma. Exp Cell Res.

[ref-15] Chen Hang, Li Molin, Huang Ping (2019). LncRNA SNHG16 Promotes Hepatocellular Carcinoma Proliferation, Migration and Invasion by Regulating miR-186 Expression. J Cancer.

[ref-16] Jing Zhao, Ye Xiaoping, Ma Xiaojie, Hu Xiangrong, Yang Wenjun, Shi Junping, Chen Gongying, Gong Ling (2020). SNGH16 regulates cell autophagy to promote Sorafenib Resistance through suppressing miR-23b-3p via sponging EGR1 in hepatocellular carcinoma. Cancer Med.

[ref-17] Ye Junfeng, Zhang Ruoyan, Du Xiaohong, Chai Wengang, Zhou Qiang (2019). Long noncoding RNA SNHG16 induces sorafenib resistance in hepatocellular carcinoma cells through sponging miR-140-5p. Onco Targets Ther.

[ref-18] Guo Zhenli, Zhang Ju, Fan Lulu, Liu Jiatao, Yu Hanqing, Li Xiaoqiu, Sun Guoping (2019). Long Noncoding RNA (lncRNA) Small Nucleolar RNA Host Gene 16 (SNHG16) Predicts Poor Prognosis and Sorafenib Resistance in Hepatocellular Carcinoma. Med Sci Monit.

[ref-19] Liu Qiuli, Gao Po, Li Qingling, Xu Chao, Qu Kai, Zhang Jie (2021). Long non-coding RNA SNHG16 as a potential biomarker in hepatocellular carcinoma: A meta-analysis. Medicine (Baltimore).

[ref-20] Guo Yongjian, Bai Mingjun, Lin Liteng, Huang Jingjun, An Yongcheng, Liang Licong, Liu Yaohong, Huang Wensou (2019). LncRNA DLEU2 aggravates the progression of hepatocellular carcinoma through binding to EZH2. Biomed Pharmacother.

[ref-21] Fu Yanchao, Li BingBing, Huang Renzheng, Ji Xia, Bai Wen-Kun (2022). Long noncoding RNA DLEU2 promotes growth and invasion of hepatocellular carcinoma by regulating miR-30a-5p/PTP4A1 axis. Pathol Res Pract.

[ref-22] Salerno Debora, Chiodo Letizia, Alfano Vincenzo, Floriot Oceane, Cottone Grazia, Paturel Alexia, Pallocca Matteo, Plissonnier Marie-Laure, Jeddari Safaa, Belloni Laura, Zeisel Mirjam, Levrero Massimo, Guerrieri Francesca (2020). Hepatitis B protein HBx binds the DLEU2 lncRNA to sustain cccDNA and host cancer-related gene transcription. Gut.

[ref-23] Zhang Mei-Jing, Yan Zhe, Qin Jing, Luo Tian-Hang, Yang Biao (2022). XIST as a valuable biomarker for prognosis and clinical parameters in diverse tumors: a comprehensive meta- and bioinformatics analysis. Neoplasma.

[ref-24] Parodi Stefano (2020). Xist noncoding RNA could act as a tumor suppressor gene in patients with classical Hodgkin's disease. J Cancer Res Ther.

[ref-25] Zhuang L. K., Yang Y. T., Ma X., Han B., Wang Z. S., Zhao Q. Y., Wu L. Q., Qu Z. Q. (2016). MicroRNA-92b promotes hepatocellular carcinoma progression by targeting Smad7 and is mediated by long non-coding RNA XIST. Cell Death Dis.

[ref-26] Brzuzan Paweł, Mazur-Marzec Hanna, Florczyk Maciej, Stefaniak Filip, Fidor Anna, Konkel Robert, Woźny Maciej (2020). Luciferase reporter assay for small-molecule inhibitors of MIR92b-3p function: Screening cyanopeptolins produced by Nostoc from the Baltic Sea. Toxicol In Vitro.

[ref-27] Pascut Devis, Hoang Minh, Nguyen Nhu N. Q., Pratama Muhammad Yogi, Tiribelli Claudio (2021). HCV Proteins Modulate the Host Cell miRNA Expression Contributing to Hepatitis C Pathogenesis and Hepatocellular Carcinoma Development. Cancers (Basel).

[ref-28] Xu C.-L., Liu Y.-H. (2022). Prognostic value of LncRNA-HOTAIR for patients with hepatocellular carcinoma: a meta-analysis. Eur Rev Med Pharmacol Sci.

[ref-29] Zhang Guangming, Wu Bin, Fu Liangyin, Liu Bin, Han Xiaoyong, Wang Jie, Zhang Yipeng, Yu Miao, Ma Haizhong, Ma Shixun, Cai Hui (2022). A pan-cancer analysis of the prognostic value of long non-coding RNA LINC00662 in human cancers. Front Genet.

[ref-30] Xu Hong, Chen Yueming, Dong Xueyan, Wang Xianjun (2018). Serum Exosomal Long Noncoding RNAs ENSG00000258332.1 and LINC00635 for the Diagnosis and Prognosis of Hepatocellular Carcinoma. Cancer Epidemiol Biomarkers Prev.

