# Supplementary material for: Inflammation-Associated Long Non-Coding RNAs (lncRNAs) in Chronic Viral Hepatitis-Associated Hepatocellular Carcinoma
Source: Turk Patoloji Derg. 2025 Jan 31;41(1):1–8. doi: 10.5146/tjpath.2024.13593 (PMC11826398; doi:10.5146/tjpath.2024.13593)
Supplement: Supplementary file 1 — Supplementary Table I PDF file supplied by authors. [file TurkPatolojiDerg-41-13593-s001.pdf]

Supplement Table I: lncRNAs included in the RT-PCR array panel.

A2ML1-AS1	HCG18	NEAT1	SENP3-EIF4A 1
ABCA11P	HNRNPU-AS1	NUTM2A-AS 1	SIK3-IT1
AC000120.7	HOTAIR	OIP5-AS1	SLC7A11-AS 1
AC007228.9	HTR4-IT1	PDXDC2P	SNHG11
MZF1-AS1	IQCF5-AS1	RMST	SNHG16
AC068196.1	JPX	LOC100287 846	SNHG5
LOC101927 156	LINC00094	RP11-282O1 8.3	SNHG7
CEP83-AS1	LINC00116	RP11-29G8.3	TP73-AS1
CROCCP2	LINC00293	RP11-325K4. 3	TUG1
CTC-444N24. 11	LINC00324	RP11-363E7. 4	XIST
CTC-487M23. 5	SNHG20	RP11-363G2. 4	ZFAS1
CTD-3185P2. 1	LINC00421	RP11-367N1 4.3	ZNRD1-AS1
DLEU2	LINC00635	RP11-38P22. 2	ACTB*
EPB41L4A-AS 1	LINC00657	RP11-399K21. 11	B2M*
ERICH1-AS1	LINC00662	RP11-473I1. 10	RPLP0*
LRRC75A-AS 1	LINC00667	RP11-473M2 0.16	RN7SK*
FGD5-AS1	LL22NC03-N 27C7.1	RP11-498C9. 15	SNORA73A*
FGF14-IT1	LOC653160	RP11-549J18. 1	HGDC*
PSMA3-AS1	LRRC37BP1	RP11-819C2 1.1	RTC*
FOXN3-AS2	MALAT1	RP11-84C13. 1	RTC*
GAS5	MCM3AP-AS 1	RP11-96D1. 10	RTC*
GAS5-AS1	MEG3	RP1-239B22. 5	PPC*
GRM5-AS1	NAV2-AS5	RP6-24A23.7	PPC*
HCG11	NCBP2-AS2	SDCBP2-AS1	PPC*

***:** Reference genes, **HGDC:** Human genomic DNA contamination well, **RTC:** Reverse transcription control, **PPC:** Positive PCR control.

Supplement Table II: Differences in lncRNA expression between the groups.*

lncRNA	G1 vs C		G2 vs C		G3 vs C		G1 vs G2		G1 vs G3		G2 vs G3	
	Fold Regulation	p	Fold Regulation	p	Fold Regulation	p	Fold Regulation	p	Fold Regulation	p	Fold Regulation	p
A2ML1-AS1			**14.26**	**0.015**							17.35	0.24
ABCA11P			**16.51**	**0.008**			**-15.45**	**0.014**			**20.68**	**0.002**
AC000120.7			14.10	0.065			-11.22	0.08			**12.99**	**0.041**
AC007228.9			**16.10**	**0.005**			**-23.02**	**0.023**			**20.53**	**0.012**
MZF1-AS1			**7.63**	**0.016**	-2.13	0.96	-7.68	0.16	2.11	0.35	**16.22**	**0.012**
AC068196.1											**12.22**	**0.017**
LOC101927156	2.17	0.14	**21.70**	**0.012**			**-10.01**	**0.028**	2.26	0.25	**22.60**	**0.005**
CEP83-AS1			**34.12**	**0.005**			**-17.08**	**0.01**			**31.65**	**0.008**
CROCCP2			15.08	0.09	-2.20	0.29	-11.69	0.12	2.84	0.08	**33.24**	**0.046**
CTC-444N24.11			**13.55**	**0.015**			-9.16	0.06			**7.16**	**0.009**
CTC-487M23.5	2.54	0.19	**24.79**	**0.013**			-9.76	0.23	2.32	0.13	**22.66**	**0.004**
CTD-3185P2.1	2.58	0.17	26.95	0.072			-10.43	0.18	2.33	0.12	**24.35**	**0.038**
DLEU2	2.15	0.23	**24.95**	**0.009**			-11.61	0.22	2.05	0.21	**23.76**	**0.004**
EPB41L4A-AS1			**8.41**	**0.014**			-4.72	0.06			**5.63**	**0.007**
ERICH1-AS1			**13.52**	**0.023**							**16.38**	**0.006**
LRRC75A-AS1			**22.60**	**0.009**			-17.28	0.16			**23.76**	**0.024**
FGD5-AS1			**9.95**	0.06			-11.41	0.25			**14.97**	**0.029**
FGF14-IT1			**15.99**	**0.011**			-11.04	0.08			**15.93**	**0.004**
PSMA3-AS1	-2.33	0.74	**10.08**	**0.03**	-3.00	0.18	**-23.45**	**0.024**			**30.25**	**0.006**
FOXN3-AS2			**5.32**	**0.02**	-3.03	0.11	-7.93	0.39	2.03	0.2	**16.13**	**0.003**
GAS5			**9.11**	**0.016**	2.17	0.15	-6.48	0.13			**4.21**	**0.012**
GAS5-AS1	2.45	0.14	**20.53**	**0.026**			-8.38	0.1			**11.08**	**0.012**
GRM5-AS1	3.18	0.18	**19.87**	**0.013**			-6.24	0.13	2.72	0.16	**16.96**	**0.005**
HCG11											**18.73**	**0.005**
HCG18			**9.77**	**0.018**			**-10.81**	**0.035**			**12.31**	**0.021**
HNRNPU-AS1	2.08	0.27	14.51	0.19	2.42	0.36	-6.98	0.29			6.00	0.13
HOTAIR	12.41	0.24	30.79	0.18	4.07	0.22	-2.48	0.52	3.05	0.35	7.56	0.19
HTR4-IT1			**19.77**	**0.014**			**-10.81**	**0.026**			**15.21**	**0.04**
IQCF5-AS1			**18.51**	**0.015**			**-16.79**	**0.024**			**18.78**	**0.006**
JPX			**12.25**	**0.015**			**-6.91**	**0.029**	2.27	0.09	**15.71**	**0.005**
LINC00094			**8.44**	**0.025**			-4.73	0.08			**9.25**	**0.01**
LINC00116			**12.44**	**0.006**			**-8.63**	**0.049**			**12.86**	**0.003**
LINC00293			**16.40**	**0.015**			-8.23	0.2			**10.80**	**0.027**
LINC00324			**20.38**	**0.012**			**-15.09**	**0.032**			13.18	0.06
SNHG20			**21.73**	**0.005**			**-21.63**	**0.008**			**12.07**	**0.001**
LINC00421	3.95	0.22	**29.11**	**0.005**			-7.37	0.22	2.71	0.18	**19.96**	**0.001**
LINC00635			49.50	0.09			-34.68	0.1	2.40	0.09	**83.08**	**0.048**
LINC00657			5.95	0.14	-2.72	0.1	-5.30	0.13	3.05	0.1	**16.16**	**0.028**
LINC00662			7.49	0.09			-7.90	0.11			**6.97**	**0.029**
LINC00667			**3.31**	**0.038**			-3.48	0.85			**5.11**	**0.009**
LL22NC03-N27C7.1			**10.16**	**0.02**			**-13.76**	**0.027**			**11.06**	**0.013**

Supplement Table II continue

LOC653160			**22.96**	**0.014**							**20.83**	**0.005**
LRRC37BP1			22.79	0.21			-19.60	0.22			24.05	0.15
MALAT1			**14.11**	**0.006**			**-16.59**	**0.008**			**18.49**	**0.001**
MCM3AP-AS1			16.64	0.06			-15.37	0.09			**17.77**	**0.023**
MEG3	2.18	0.25	**18.16**	**0.016**			**-8.34**	**0.024**			**13.10**	**0.008**
NAV2-AS5			**12.84**	**0.016**			**-16.10**	**0.025**			**13.85**	**0.005**
NCBP2-AS2			9.45	0.14			-17.35	0.23			15.57	0.09
NEAT1			3.88	0.17			-2.69	0.57	2.13	0.23	**5.73**	**0.008**
NUTM2A-AS1	**-2.08**	0.48	7.81	0.31	**-2.20**	0.83	**-16.22**	0.35			17.18	0.24
OIP5-AS1	**-2.36**	0.79	**6.86**	**0.031**	**-2.35**	0.24	**-16.18**	**0.024**			**16.12**	**0.005**
PDXDC2P	**2.21**	0.18	**21.66**	**0.011**			-9.80	0.057			**16.87**	**0.003**
RMST			**35.35**	**0.006**			**-19.88**	**0.013**			**39.01**	**0.001**
LOC100287846	**2.73**	0.17	**23.51**	**0.012**			**-8.63**	**0.024**	3.40	0.09	**29.34**	**0.003**
RP11-282O18.3			**32.15**	**0.015**			**-26.76**	**0.019**			**38.49**	**0.005**
RP11-29G8.3			7.03	0.059			-8.06	0.08			**9.75**	**0.037**
RP11-325K4.3			**5.43**	**0.048**			-6.54	0.14			**4.96**	**0.023**
RP11-363E7.4											**18.60**	**0.005**
RP11-363G2.4			**22.35**	**0.014**			**-16.42**	**0.021**			**19.67**	**0.005**
RP11-367N14.3			**26.27**	**0.009**			**-19.30**	**0.016**			**26.22**	**0.002**
RP11-38P22.2			49.82	0.37			-28.97	0.37			42.91	0.30
RP11-399K21.11	**4.79**	0.33	18.17	0.21			-3.79	0.25	5.98	0.25	22.69	0.15
RP11-473I1.10			**12.02**	**0.018**	**-2.17**	0.28	-10.66	0.059	**2.45**	**0.039**	**26.08**	**0.004**
RP11-473M20.16	**2.04**	0.15	**31.33**	**0.07**			-15.37	0.09	2.34	0.08	**35.91**	**0.041**
RP11-498C9.15	**3.01**	0.16	**27.44**	**0.011**			-9.11	0.07	2.90	0.1	**26.41**	**0.003**
RP11-549J18.1			**27.06**	**0.023**			**-25.76**	**0.036**			**48.00**	**0.008**
RP11-819C21.1			10.51	0.77	**-2.59**	0.25	-7.85	0.08	**3.47**	**0.043**	**27.27**	**0.016**
RP11-84C13.1			**13.85**	**0.01**			**-13.15**	**0.016**			**25.12**	**0.002**
RP11-96D1.10			**20.55**	**0.01**			**-19.03**	**0.023**			**29.68**	**0.004**
RP1-239B22.5			**22.36**	**0.013**			**-17.36**	**0.021**			**27.79**	**0.004**
RP6-24A23.7			**27.86**	**0.008**			**-24.05**	**0.013**			**33.18**	**0.002**
SDCBP2-AS1	**3.51**	0.13	27.28	0.12			-7.76	0.21	4.35	0.08	33.76	0.072
SENP3-EIF4A1			**20.88**	**0.014**			**-12.68**	**0.024**	2.20	0.16	**27.93**	**0.004**
SIK3-IT1			**12.81**	**0.012**			**-10.15**	**0.023**	2.25	0.08	**22.82**	**0.003**
SLC7A11-AS1			**24.41**	**0.034**			-16.88	0.054			**25.68**	**0.017**
SNHG11			**4.91**	**0.024**	**-4.38**	**0.028**	-7.76	0.053	2.78	0.06	**21.54**	**0.003**
SNHG16			**8.65**	**0.006**			-8.33	0.09			**13.85**	**0.001**
SNHG5	**2.24**	0.16	**4.55**	0.14			-2.03	0.97	3.84	0.08	7.80	0.070727
SNHG7			**10.52**	**0.024**			**-8.78**	**0.044**			**12.36**	**0.011**
TP73-AS1			**24.34**	**0.009**			**-26.39**	**0.014**			**23.05**	**0.002**
TUG1			**7.50**	0.2			-12.00	0.09			**9.88**	**0.036**
XIST	**-2.96**	0.31	**3.80**	0.281	**-2.33**	**0.047**	-11.27	0.74			**8.85**	**0.003**
ZFAS1	**2.20**	0.07	**3.83**	0.14					3.40	0.95	5.92	0.86
ZNRD1-AS1	**3.21**	0.34	**34.59**	0.11	**2.36**	0.55	-10.79	0.43			14.64	0.45

Group 1 (G1): Tumor samples, Group 2 (G2): Peritumoral cirrhotic liver parenchyma, Group 3 (G3): Cirrhotic chronic viral hepatitis samples, and Control group (C): Normal liver tissue samples.
